# *piggyBac* Transposon plus Insulators Overcome Epigenetic Silencing to Provide for Stable Signaling Pathway Reporter Cell Lines

**DOI:** 10.1371/journal.pone.0085494

**Published:** 2013-12-20

**Authors:** Valeri V. Mossine, James K. Waters, Mark Hannink, Thomas P. Mawhinney

**Affiliations:** 1 Department of Biochemistry, University of Missouri, Columbia, Missouri, United States of America; 2 Experiment Station Chemical Labs, University of Missouri, Columbia, Missouri, United States of America; 3 Department of Child Health, University of Missouri, Columbia, Missouri, United States of America; Institut National de la Santé et de la Recherche Médicale, France

## Abstract

Genetically modified hematopoietic progenitors represent an important testing platform for a variety of cell-based therapies, pharmaceuticals, diagnostics and other applications. Stable expression of a transfected gene of interest in the cells is often obstructed by its silencing. DNA transposons offer an attractive non-viral alternative of transgene integration into the host genome, but their broad applicability to leukocytes and other “transgene unfriendly” cells has not been fully demonstrated. Here we assess stability of *piggyBac* transposon-based reporter expression in murine prostate adenocarcinoma TRAMP-C2, human monocyte THP-1 and erythroleukemia K562 cell lines, along with macrophages and dendritic cells (DCs) that have differentiated from the THP-1 transfects. The most efficient and stable reporter activity was observed for combinations of the transposon inverted terminal repeats and one 5’- or two cHS4 core insulators flanking a green fluorescent protein reporter construct, with no detectable silencing over 10 months of continuous cell culture in absence of any selective pressure. In monocytic THP-1 cells, the functional activity of luciferase reporters for NF-κB, Nrf2, or HIF-1α has not decreased over time and was retained following differentiation into macrophages and DCs, as well. These results imply *pB* as a versatile tool for gene integration in monocytic cells in general, and as a convenient access route to DC-based signaling pathway reporters suitable for high-throughput assays, in particular.

## Introduction

Signaling pathway reporters are defined as synthetic DNA sequences incorporating a *cis*-regulatory transcriptional response element (TRE) followed by a minimal promoter and a reporter gene [1]. The significance of signaling pathway reporter technology for cell biology cannot be overestimated. A diverse range of applications in such areas as drug discovery [2,3], toxicology or environmental surveys [4,5], which employ the reporter transgenic bacteria, eukaryotic cell lines, or animals, have been developed in recent years. 

Blood-borne cells, such as dendritic cells (DCs) or their monocyte progenitors, represent an attractive resource for developing various human cell-based assays [6,7], due to the strong responses to immunostimulants and convenience of their isolation and genomic manipulation *ex vivo*. Dendritic cells are professional antigen-presenting cells and are key regulators of the inflammatory status in response to the microbiota, environmental stress and pathogen invasions [8,9]. Mature DCs normally reside in epithelia and thus are well suited for development of reporter-based assays for potential skin, lung, or mucosal allergens. One current limitation is the lack of a robust approach to generation of stable DC-based reporters. Ideally, the reporter cells would represent immortalized lines stably expressing both stimulated and marker reporter genes. Mature DCs do not proliferate, but could be differentiated *in vitro* from monocytic precursors [10]. However, previous attempts to use isolated blood monocytes for securing stable reporter transgene activity in differentiated DCs were not successful [11]. An alternative to isolated monocytes, human monocytic leukemia line THP-1 is one of the widely used models for monocytic progeny of phagocytic cells, such as macrophages [12,13]. In addition, high relevance of THP-1 monocytes to isolated DCs has been verified in skin sensitization assays [14] using qPCR analysis of representative marker genes. Hence, we asked whether pathway reporter DCs could be reliably generated from THP-1 stably expressing respective reporter genes. 

Stable expression of transgenes in human cells has received a significant effort from both academia and pharma, due to a wide variety of potential applications for genetically modified cells [15,16]. Regulation of gene expression in eukaryotes depends on multiple *cis*- and trans-acting factors, including spatial organization of chromatin, nucleosomal positioning on regulatory sequences, and covalent modifications of DNA nucleotides and histones. Random exogenous polynucleotide integration into the host DNA often leads to the transgene silencing [17]. To achieve continuous expression of the target transgene, a number of approaches have been developed in recent years [18]. Although the most popular systems currently used for gene integration into mammalian cells are viral vectors, they *per se* do not provide transgene protection from silencing, while their cargo size limitations and safety concerns have prompted an increase in efforts to develop efficient non-viral gene delivery and protection tools [19]. The task has been addressed by the establishment of “humanized” transposons and insulators. 

The *piggyBac* transposon (*pB*) was initially identified in insects over three decades ago, but has been introduced into the gene technology field only recently [20]. The *pB* transposon has a number of advantages over traditional transfection and viral gene delivery approaches. First, the transgene integration into the target genome is not spontaneous, but is mediated by co-transfected *pB* transposase (*pB*ase) in a “cut-and-paste” fashion. Second, the insertion sites tend to localize in transcription-active areas rich in CpG islands. Finally, the size of *pB* transposon cargo gene (over 200 kb [21]) dramatically exceeds the limit of viral particles (5-10 kb). 

Insulators are *cis*-regulatory elements that can block improper gene activation or heterochromatin propagation imposed by remote enhancers and silencers. One of the best studied insulators is chicken hypersensitive site-4 (cHS4) boundary element at the β-globin locus, which acts by recruiting histone acetyl transferases and hindering DNA methylation of the transgene promoter [22]. In addition, cHS4 may act as an enhancer blocker, although by a different mechanism. Insulators thus have been successfully applied for stabilization of transgenes, such as those delivered by viral vectors [22,23].

In this study, we have assessed whether *pB* transposon mediated transfer of insulated reporter gene series into THP-1 cells could provide for stably altered monocytic cell lines capable of further cell differentiation into tissue-specific macrophages or DCs with the reporter activities preserved. To the best of our knowledge, this approach enabled, for the first time, a facile generation of DC-based signaling pathway reporters suitable for high-throughput assays. 

## Results

The overall efficiency of stable transgene expression depends on the gene delivery, integration into the host genome, and epigenetic regulation of its promoters due to the positional and silencing/ enhancing effects. Monocyte-derived cells are well equipped for recognition and clearance of foreign DNA [24], and are resistant to stable transfections [15]. Although there are a number of reports claiming successful transient transfection of reporter DNA into THP-1 and other monocytes, the vectors most often described in the literature, such as pmaxGFP [25], are relatively small, and the reports do not normally elaborate on efficiency of the vector incorporation into the host genome. In our hands, THP-1 resisted most of tested transfection protocols, with the exception of nucleofection when performed specifically with pmaxGFP. The green fluorescent protein (GFP) expression in the nucleofected cells, however, was not observable after 1-2 weeks, due to the silencing or cell death. In addition, monocytes are notoriously sensitive to manipulations, and our attempts to enrich transfected monocytes by flow cytometry or cloning were met with a mixed success owing to very low cell survival rates. We, therefore, decided to turn to different cell lines which would display a significant level of transgene silencing, but would be tolerant to conventional transfection reagents and DNA vectors, as well as enrichment protocols.

### Flanking a GFP gene with insulators and pB ITRs overcomes the transgene silencing in TRAMP-C2 and K562 cells

Transgenic mouse prostate adenocarcinoma (TRAMP) is a popular model for prostate cancer studies, both in vitro and in vivo [26,27]. Reports on stably transfected TRAMP cell lines are scarce, while the cell line’s propensity to efficiently enforce downregulation on many genes through chromatin hypermethylation is well documented [28,29]. When we transfected the TRAMP-C2 line with a standard pEGFP-C1 reporter plasmid and then attempted to enrich stably transfected cells by either G418-mediated selection or flow cytometric sorting, only about 3-5% of the selected cells remained fluorescent (Figure S1A). A similar effect was observed when a GFP reporter transgene was stably integrated into the chromatin of TRAMP-C2 cells using a lentiviral vector (Figure S1B). The cells displayed a heterogeneous pattern of GFP expression in growing colonies, as well (Figure S2). In a striking contrast, when TRAMP-C2 cells were transfected with a transposon-based GFP-encoding vector (pB513B-1) along with a *piggyBac* transposase (*pB*ase) plasmid and sorted by flow cytometry, the GFP-positive cells have uniformly retained GFP expression and puromycin resistance (Figure S1C). 

**Figure 1:**
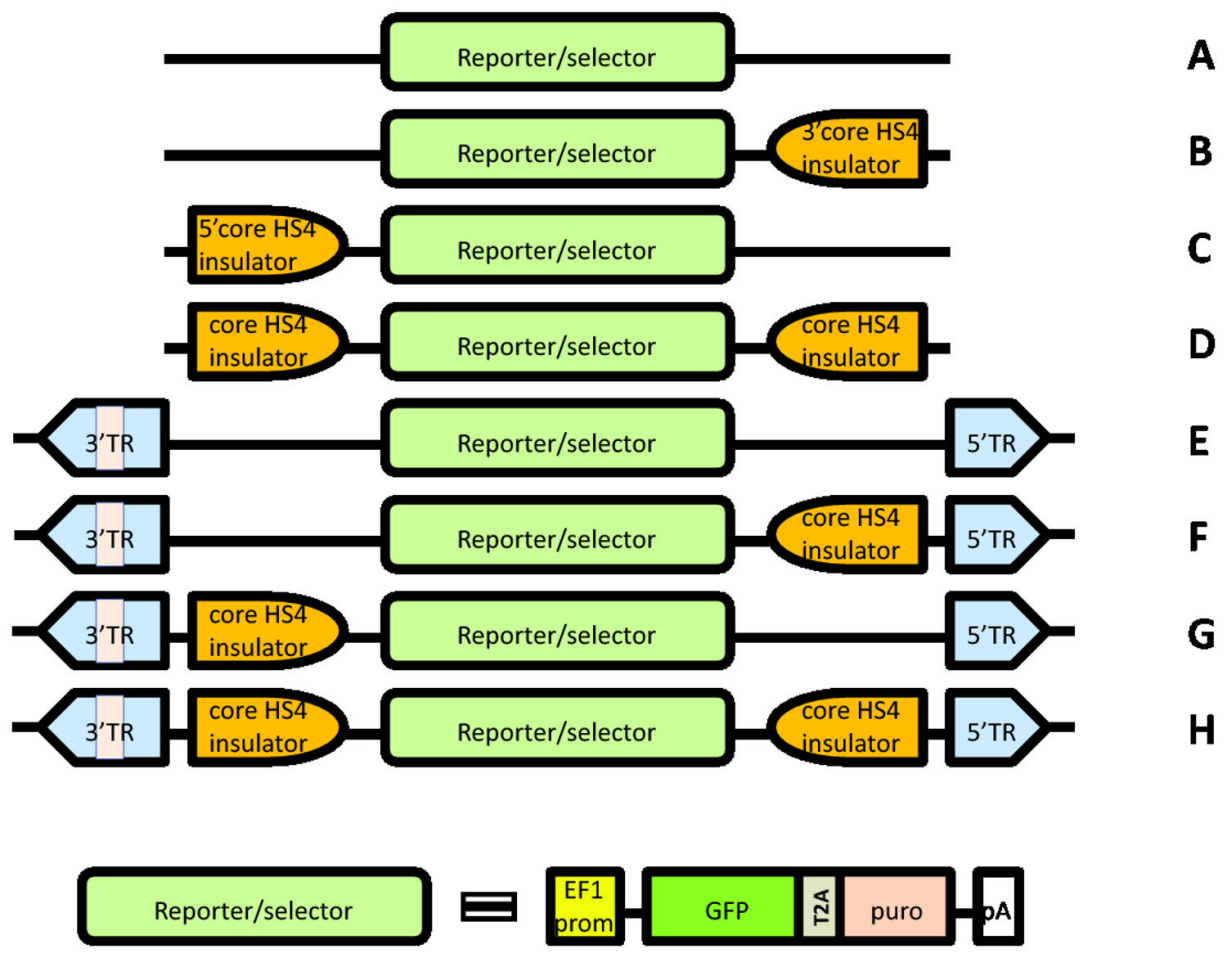
Schematic representation of GFP reporter constructs. The constructs **A** through **H** were inserted into pSMART, resulting in pS**A** through pS**H** plasmids, respectively.

**Figure 2:**
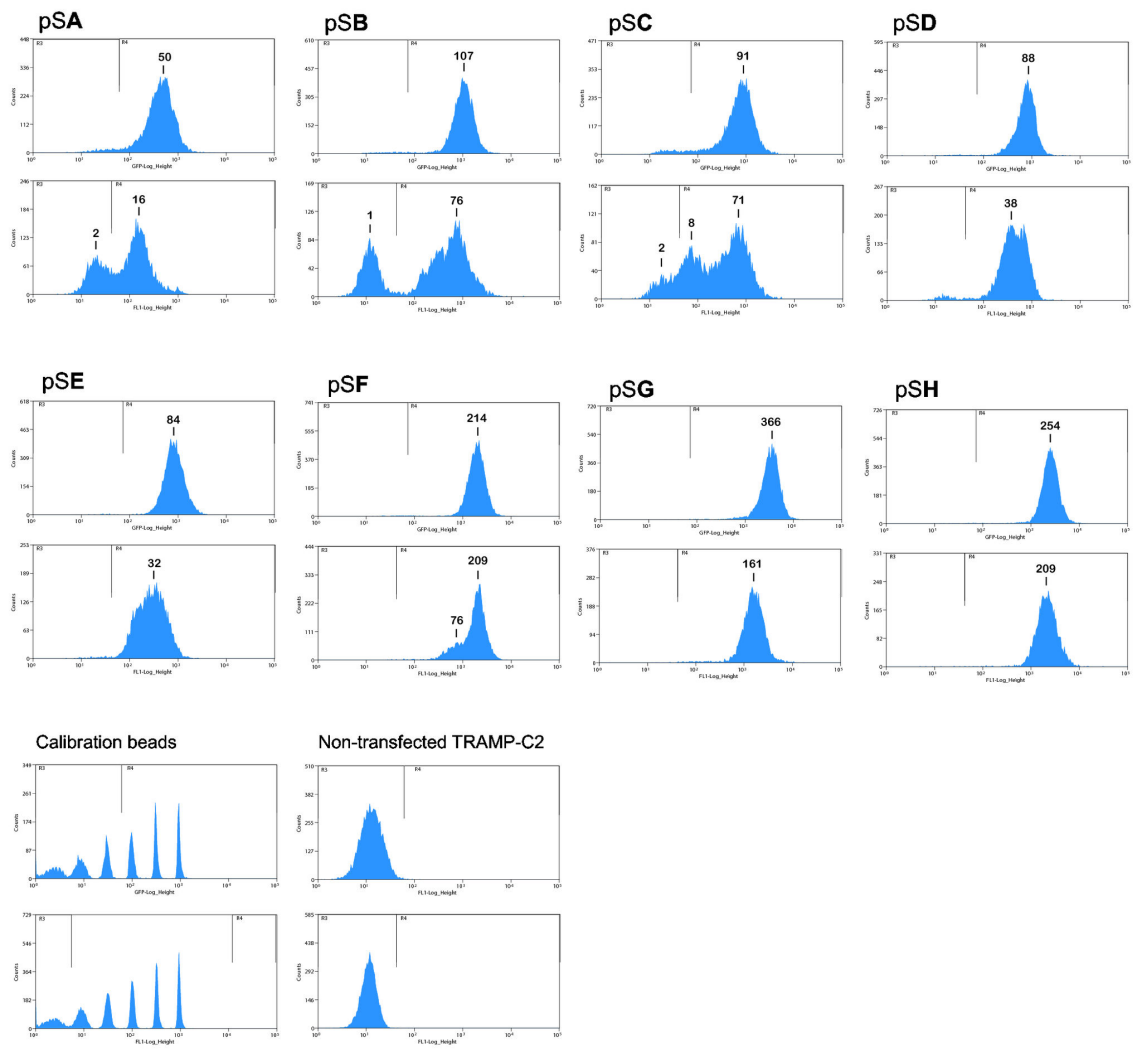
*piggyBac* transposon-based, insulated reporters are stably expressed in TRAMP-C2 line. Vectors pS**A** through pS**H** were co-transfected with the Super *pB* transposase plasmid into TRAMP-C2 cells. The transfects went through several rounds of FAC sorting, until the maximum fluorescence have been achieved (the upper histogram in each pair). The sorted cells were then let to proliferate without any selection and were subcultured twice a week. The lower histogram in each pair represents FACS analyses of the cells at the end of the 4-month term. Immediately before the analyses, the FACS instrument settings were calibrated against Sphero Rainbow Beads. Median fluorescence intensities in the transfected cells are normalized for the non-transfected control TRAMP-C2 autofluorescence.

pB513B-1 contains both *pB* transposon inverted terminal repeat (ITR) recognition sites and two core HS4 insulators flanking the GFP reporter/ puromycin resistance sequence (Figure 1H). To determine the individual roles of *pB* and HS4 in the stabilization of reporter activity in TRAMP-C2 cells, a systematic analysis was performed. The CMV/MCS was excised from the transposon and the remaining elements were reassembled in a barebone pSMART plasmid (constructs **A** through **H** in Figure 1). TRAMP-C2 were transfected with plasmids carrying **A** through **H** (pS**A** through pS**H**), along with the *pB*ase plasmid. All transfects were enriched by fluorescence-activated cell sorting (FACS) in 5 to 6 biweekly sort rounds, until 100% fluorescent, and then left to proliferate for 4 months without any further selection. The flow cytometry analysis is presented in Figure 2 and clearly shows two patterns. At the beginning of the 4-month period (the upper histograms in the pairs), the fluorescence intensity was the lowest (median 50-fold over autofluorescence in the non-transfects) in cells transfected with pS**A**, which lacks both transposon and insulator sequences. Cells transfected with pS**B** through pS**E**, that is, possessing either *pB* or at least one cHS4 insulator, formed a group with an intermediate fluorescence intensity (median 84 to 107-fold). The strongest median fluorescence (214 to 366-fold) was displayed by cells transfected with vectors pS**F** through pS**H**, containing both *pB* and at least one cHS4. At the end of the 4-month run (the lower histograms in the pairs, Figure 2), there was a significant number of non-fluorescent cells in TRAMP-C2 transfected with pS**A** through pS**C**. In the cells transfected with vectors containing both insulators (pS**D**), transposon only (pS**E**), or transposon plus one insulator (pS**F** and pS**G**), we observed a shift in the cell populations towards 2 to 3-fold decrease in median GFP expression, but no cells with a complete loss of the reporter. In contrast, there was only about 20% decrease in median GFP expression in cells that have the construct **H**, with both insulators flanking the GFP reporter gene, that were inserted into the chromatin by *pB* transposition.

Human erythroleukemia K562 line is another well-established example of transgene silencing. It has been reported to efficiently reduce expression of stably transfected GFP, presumably due to both epigenetic silencing and excision of the transgene from the integration sites in the host genome [30]. When K562 cells were transfected with pS**A**, pS**D**, or pS**H** and then FACS-selected for the uniformity of fluorescence, the resulting stable transfects displayed a GFP expression intensity and stability pattern, in the order pS**H**>pS**D**>pS**A** (Figure S3), which is similar to the one observed in TRAMP-C2 cells transfected with the respective vectors. 

### Expression of TRE-luciferase-GFP reporter constructs is stable in monocytic THP-1

Encouraged by the successful stabilization of GFP transgene expression in TRAMP-C2 and K562 cells, we then assessed the ability of the transposon vector pS**H** to provide for stable GFP expression in THP-1 cells. We have succeeded with the task by employing a non-toxic transfection reagent, GeneIn, as a suitable vehicle for pS**H** and *pB* transposase plasmids co-delivery into the monocytic cells (Table S1). Having achieved stable transfection of transposon-based GFP-only vectors into THP-1, we next asked whether significantly larger *pB* transposons, carrying additional luciferase reporters, could be introduced into THP-1 genome as well.

**Figure 3:**
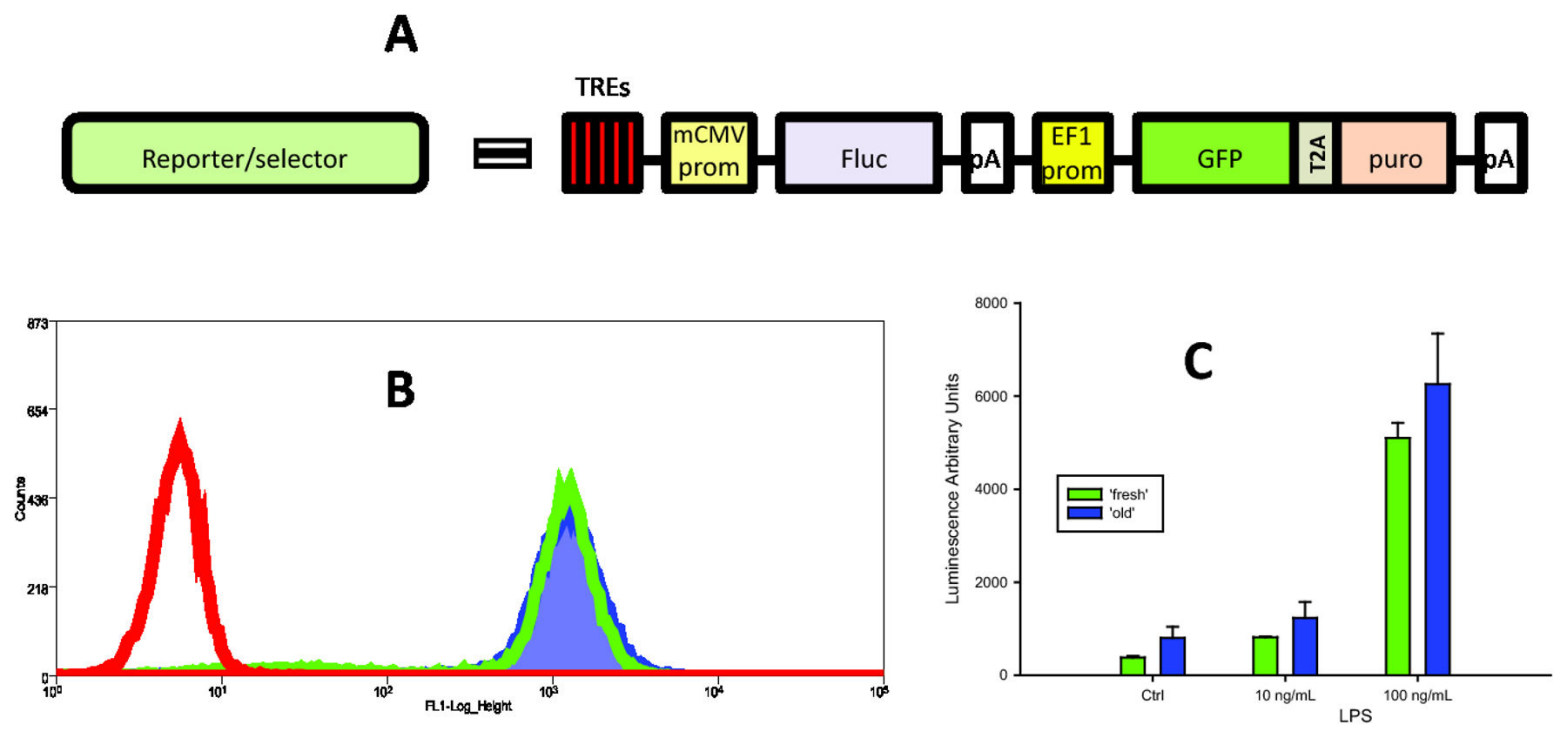
Stable transfection of monocytic THP-1 with pB-based, insulated NF-κB reporter vector pTR01F. (**A**) Schematic representation of the pathway reporter (See also Figure S4) transposon, insulated as in construct **H** (Fig 1A). (**B**) FACS analysis of GFP expression in THP-1 (non-fluorescent control, red diagram), the transfect THP.R01F freshly thawed from a cryopreservation (green), and THP.R01F that was cultured continuously for 10 months without any selection (blue). (**C**) Relative activity of NF-κB reporter in ‘fresh’ and ‘old’ THP.R01F batches in response to LPS treatment.

The sequences that are encoding for multiple TREs specific for NF-κB, HIF-1α, and Nrf2 and are followed by a minimal CMV promoter for the firefly luciferase gene have been inserted into the construct **H**, thus affording reporter vectors for NF-κB, hypoxia-induced, and antioxidant/electrophile signaling pathways, respectively (Figures 3A and S4). These products are abbreviated accordingly as pTR01F, pTR03F, and pTR05F, while respective stable cell transfect names are denoted by the extensions R01F, R03F, or R05F. 

Thus, the lines THP.R01F, THP.R03F, and THP.R05F were generated following co-transfection of THP-1 with the respective reporter transposon and *pB* transposase plasmids, followed by puromycin-driven selection. All puromycin-resistant cells were expressing GFP and did not require any further selection. To verify the reporter transgene stability, we have cultured THP.R01F continuously for 10 months, without any selection pressure. The FACS analysis showed no decrease in GFP expression and NF-κB reporter activity in this “old” culture, when compared to the original batch of THP.R01F freshly thawed from cryopreservation (Figure 3B). As illustrated in Figure 3C, when both batches were treated with an NF-κB activator, lipopolysaccharide (LPS) from *E.coli*, there was no decrease in luciferase expression in the “old” THP.R01F culture, as well. Similar reporter stability was observed in THP.R03F and THP.R05F lines. 

### Differentiation of the monocyte reporters into mature macrophages and dendritic cells retains the reporter activities

**Figure 4:**
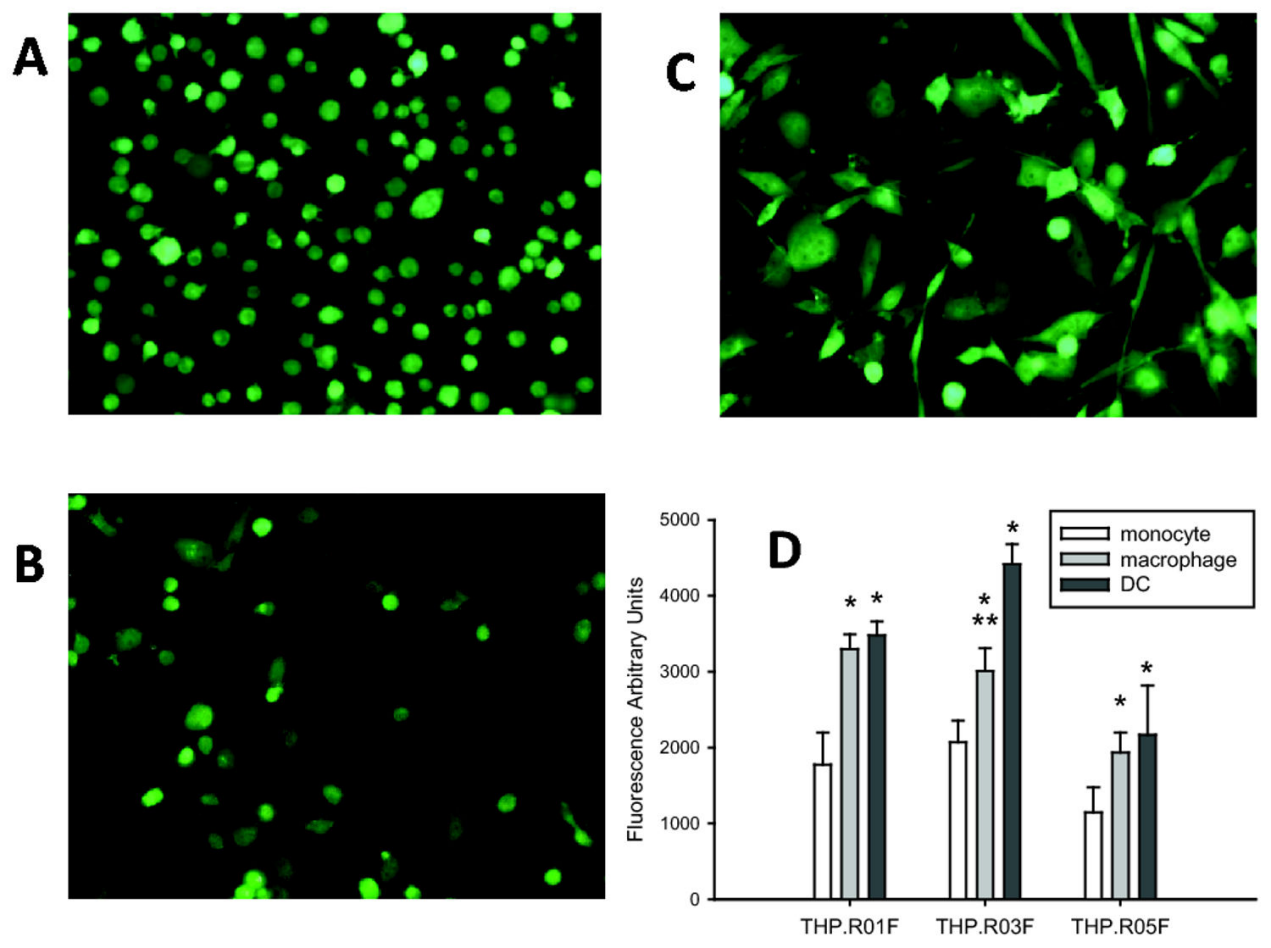
Differentiation of THP.R01F monocytes does not interrupt with the GFP reporter activity. Monocytic THP.R01F (**A**) were transformed into macrophages (**B**) upon treatment with 100 ng/mL PMA for 3 days. Alternatively, treatment of the monocytes with 100 ng/mL GM-CSF and 200 ng/mL IL-4 for 16 hours, followed by 20 ng/mL TNFα and 200 ng/mL ionomycin, produced mature dendritic cells (**C**) within next two days. 96-Well assay plates were seeded with 2×10^4^ THP.R0nF cells (n = 1, 3, or 5 for NF-κB, HIF-1α, or Nrf2 reporters, respectively) per well, six wells per cell line. The cells were differentiated as above, lysed, and relative fluorescence of the lysates was measured (**D**). The bars and error bars are fluorescence means ± SD, n = 6; statistical analysis: one-way ANOVA, with pairwise multiple comparisons by Holm-Sidak test. Statistical significance *: compared to white bar; **: compared to dark grey bar; in all cases, *P*<0.01.

To determine whether THP.R0nF (n = 1, 3, or 5) have retained the potential of immature phagocytic progenitors, we performed differentiation of the monocyte transfects into mature macrophages or DCs. When THP.R0nF were treated with phorbol 12-myristate 13-acetate (PMA) for 2 days following a standard differentiation protocol, all cells became tightly adherent, with characteristic macrophage morphology (Figure 4B). However, exposure of the monocytic transfects to DC differentiating factors GM-CSF and IL-4, followed by TNF-α/ ionomycin treatment as described by Berges et al. [31], produced DCs (Figure 4C), also within only 2 days. Importantly, these treatments afforded for 100% adherent cells with no signs of cytotoxicity, provided PMA or TNF-α/ ionomycin media were removed after the differentiation was complete. All differentiated macrophages and DCs displayed strong GFP expression, comparable with their monocytic progenitors (Figure 4D).

**Figure 5:**
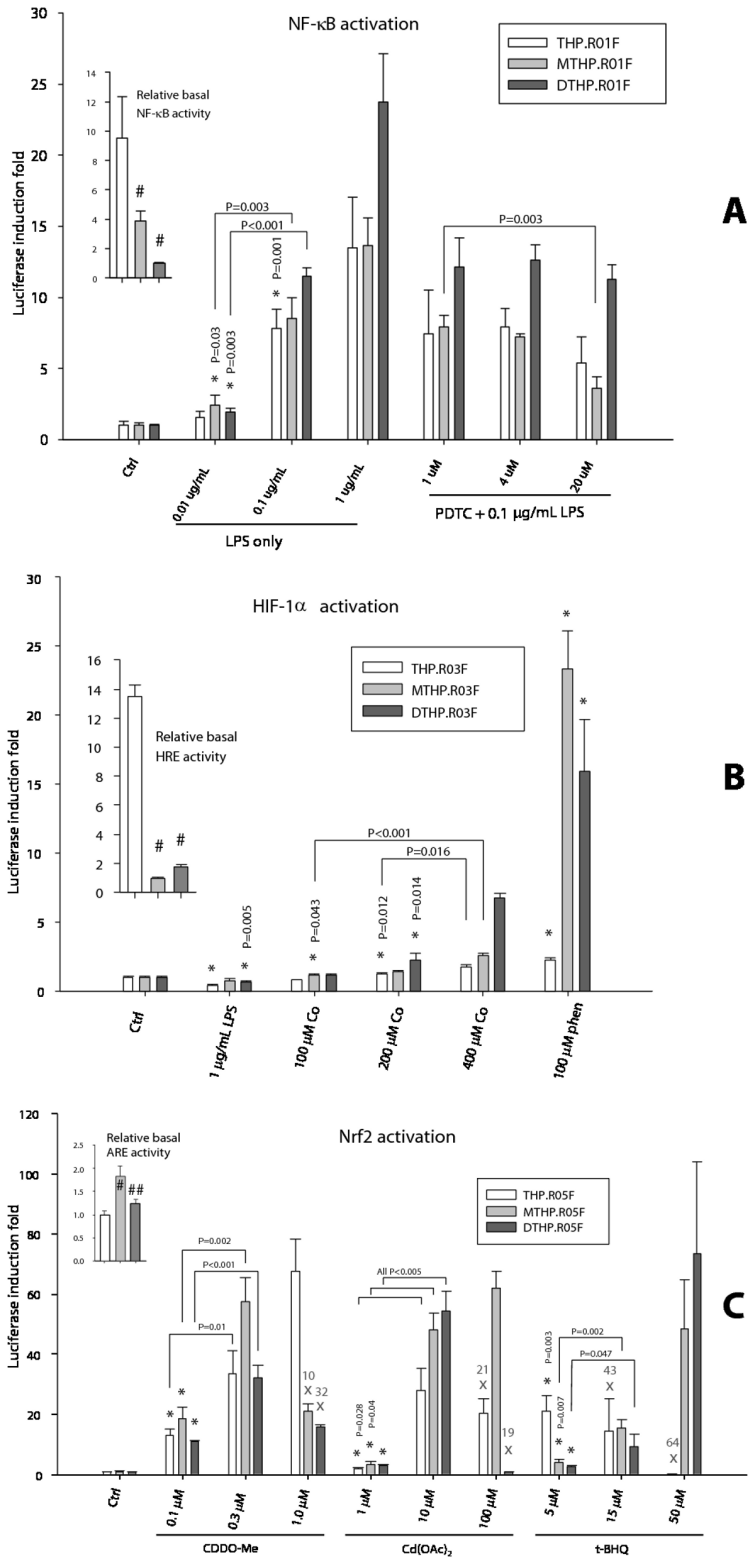
Differentiation of monocytic THP.R0nF pathway reporters into macrophages or dendritic cells retains the reporter activity. (**A**) Monocytes THP.R01F were differentiated into macrophages (MTHP.R01F) and DCs (DTHP.R01F) and then treated, in triplicates, for 4 hours with LPS only or in presence of NF-κB activation inhibitor PDTC. (**B**) THP.R03F, MTHP.R03F, and DTHP.R03F cells were treated, in triplicates, for 16 hours with LPS or hypoxia mimics CoCl_2_ and o-phenanthroline. (**C**) The ARE/EpRE activity in THP.R05F, MTHP.R05F, and DTHP.R05F cells treated, in triplicates, for 16 hours with inducers of Nrf2. The **X**’s indicate treatments too toxic to cells and percentages of cell fluorescence loss due to apoptosis/death. Fluorescence of lysates of all treated cells was used as a cell enumeration surrogate. The luciferase activity induction folds for each cell type were normalized to the respective controls. The relative basal TF activities in the untreated controls were normalized to the lowest value and are shown in the insets. The bars and error bars are normalized luminescence means ± SD, n = 3. Statistical analysis in the insets: one-way ANOVA, with pairwise multiple comparisons by Holm-Sidak test. Statistical significance #: compared to white bar; ##: compared to light grey bar; in all cases, P<0.01. Statistical analysis in the main charts: pairwise comparisons by an unpaired **t**-test. Statistical significance *: the lowest dose treatment compared to respective control; P≤0.001, if not indicated otherwise.

Next, inducible reporter luciferase activity for each signaling pathway reporter was compared between the monocytes and respective differentiated macrophages and DCs. Aiming at development of practical high-throughput assays, the comparative studies were carried out in a 96-well plate format. According to a protocol which we followed, THP.R0nF were seeded into the inner wells of 96-well plates, at 2×10^4^ cells/well, and some were treated with the differentiating agents for 2 days in serum-free media. The differentiation media were replaced with an assay medium to adapt for next 16-24 h. Following the adaptation period, specific pathway stimulating agents were added to the monocyte, macrophage and DC reporters for desired periods of time. Then treated and untreated cells were lysed, and fluorescence of GFP in the lysates was measured in a capacity of an internal cell number surrogate, in order to evaluate the cell proliferation/death rate and to normalize the following luminescence data. Finally, the luciferin substrate was added to the lysates, and the luminescence in wells was measured. The assay data are compiled in Figure 5.

As follows from Figure 5A inset, the relative basal NF-κB activity, determined as luminescence normalized to the fluorescence readings in the unstimulated wells, was the highest in THP.R01F monocytes and the lowest in the DCs (DTHP.R01F), a 10-fold difference. When stimulated with a bacterial LPS, the luciferase activity increased in all cells in a dose-dependent manner, reaching 25-fold induction in DCs and 15-fold increase in monocytes and macrophages for the highest tested LPS dose. 

The relative basal activity of the hypoxia-inducible pathway was again the highest in the monocytic THP.R03F, reaching 13-fold difference with the minimally active macrophages (MTHP.R03F, Figure 5B inset). Upon stimulation with hypoxia mimics CoCl_2_ and o-phenanthroline, however, the strongest response was detected in the macrophages, 24-fold increase, and DCs (16-fold), also in a dose-dependent manner (Figure 5B). Interestingly, the NF-κB activator LPS has inhibited the basal HIF-1α activity in all cells.

The unstimulated THP.R05F monocytes showed antioxidant/electrophile pathway activity that was similar to the DCs’, while the MTHP.R05F macrophages demonstrated nearly twofold higher level of the basal activity (Figure 5C inset). All cells responded strongly to specific inducers of the Nrf2-mediated antioxidant/electrophile response element (ARE/EpRE) activity, reaching 60 to 70-fold induction at the maximum. All three standard ARE/EpRE stimulators, a Michael addition acceptor triterpenoid CDDO-Me, heavy metal cadmium, and redox active tert-butyl hydroquinone (t-BHQ), showed cytotoxicity at elevated doses. The cytotoxicity was cell type specific. Thus, the monocytes were most affected by t-BHQ cytotoxicity but were insensitive to CDDO-Me; the macrophages were inhibited by CDDO-Me only, while DCs viability was affected by both the triterpenoid and cadmium (Figure 5C).

We have also prepared stable pathway reporter lines based on TRAMP-C2 (abbreviated as TC2) and K562. These were tested in presence of the NF-κB, HIF-1α, or Nrf2 activators, and the results are depicted in Figures S5-S8. All tested reporter lines displayed strong responses to the specific stimulating agents, whereby the activation magnitudes were cell-specific. Thus, K562.R01F was indifferent to LPS treatment, as compared to its strong responses to TNFα and IL-1β. TC2.R05F were much less sensitive to inducers of ARE/EpRE as compared to the THP-based Nrf2 reporter cell lines, while HIF-1α-activating potential of a powerful hypoxia mimic, o-phenanthroline, was only marginal in THP.R03F.

## Discussion

In this study, we employed *piggyBac* transposon-mediated gene transfer to successfully introduce functional signaling pathway reporters into a monocytic line THP-1 and the monocyte-derived macrophages and dendritic cells. Although transposons have previously been used for gene delivery into hematopoietic stem cells and lymphocytes, this work deals with monocyte-derived cells and thus widens the scope of potential applications for the transposon technology in immunology related areas. For example, stabilization of hypoxia-inducible therapeutic gene expression in tumor-infiltrating macrophages could potentially aid in development of cell-based antitumor therapies [15,32]. Another particular application for transposon-based, insulated TF-reporters is generation of reporter DCs for high-throughput assays that could become an important *in vitro* alternative for environmental allergen assays, such as those that currently use animals for skin or mucosa sensitization [7,33]. 

In addition to *pB* employed in this work, there are two other transposon systems currently being actively developed as potential candidates for therapeutic gene administration into the human genome: *Sleeping Beauty*, and *Tol2*. A few publications offer comparisons of the three transposons head-to-head [34,35], but the field is very dynamic and new, more powerful, transposase modifications are being rapidly developed [36,37], thus quickly outdating any comparative studies. Although our results obtained with *pB* may be well achievable with the other “humanized” transposons, establishing the relative transposon efficiency was not critically significant for the purpose of this work. More importantly, we have opted in favor of a transposon system over virus-based vectors following a failure of the lentivirus-mediated gene transduction to provide for stable expression of a GFP reporter in TRAMP-C2 (Figure S1B). In addition, viral particles have a relatively low size cap on cargo DNA load, which is about 5 kb for the currently most popular adeno-associated virus (AAV), for example. Transposons can operate with much larger DNA loads [21], which suited our goal of developing double reporter constructs with the size of 6 kb, such as in pTR01F, and up.

Since incorporation of a transgene into a host genome does not guarantee stable expression of the transgene *per se*, even though *pB* insertions are biased towards actively transcribed loci [38], we have tested an additional means of epigenetic stabilization for the reporter expression in TRAMP-C2 cells. The barrier insulators, such as the most commonly used HS4 core sequence derived from the chicken β-globin gene cluster, are able to resist heterochromatin spread and subsequent silencing of an adjoining DNA region [22]. Indeed, a combination of two flanking HS4 core insulators in the non-transposable construct **D** provided for better stability of GFP expression in TRAMP-C2, with loss of about one half of highly fluorescent population after 4 months, as compared to the lacking cHS4 transposon pS**E**-transfected cells, which showed a complete shift to decreased GFP expression within the same period of time (Figure 2). A combination of two HS4 insulators and *pB* in pS**H**-transfected cells, on the other hand, provided for a maximum in both the stability and efficiency of GFP expression, after the continuous 4-month culturing.

In addition to tracking a transgene expression, constitutive production of GFP in the signaling pathway reporter cells can be useful for development of high-throughput assays, since GFP fluorescence can be used as an internal surrogate for relative cell enumeration [39] done simultaneously with measuring luciferase activity in cell lysates, thus enabling protocols for 96- or even 384-well plate formats. The copepod GFP employed in our reporter constructs is well suited for such assays, since it is “destabilized” and, unlike the EGFP family, does not over-accumulate intracellularly and does not affect important signaling pathways such as NF-κB [40]. Hence, a combination of a signaling pathway reporter construct, such as TRE-mCMV-luc, and a cell tracking reporter, such as cGFP, under protection of insulators and transposon, should provide for simplification and an extended utility of cell-based screening protocols.

Indeed, the initial testing of the TF-reporting activities in the reporter THP-derived cells proved the assay is easy to set, relatively rapid and reproducible. The reporter cells responded strongly and dose-dependently to specific inducers of NF-κB, HIF-1α, or Nrf2 transcription factor activity. The assay, therefore, allows for simultaneous assessment of signaling pathway activity in monocytic cells and monocyte-derived macrophages and DCs. Moreover, the availability of stable THP-based signaling pathway reporters may allow for developing assays employing other tissue specific phagocytes, such as microglia or osteoclasts, since these cell types can be readily prepared from monocytic precursors in vitro [41,42]. The equally successful generation and testing of stable signaling pathway reporters based on TRAMP-C2 and K562 cell lines indicates a broad, if not universal, applicability of the transposon plus insulators approach when overcoming epigenetic transgene silencing is required.

## Materials and Methods

Cell differentiation agents human GM-CSF and IL-4 were from Shenandoah Biotech; PMA, ionomycin, lipopolysaccharide (*E. coli* 026-B6), cadmium acetate, cobalt chloride, nickel chloride, oltipraz, tert-butylhydroquinone, o-phenanthroline were from Sigma-Aldrich, human IL-1β and murine TNFα were purchased from PeproTech, while CDDO-Me was a gift from TransTech Pharma.

### Cell culture

TRAMP-C2, K562, and THP-1 cell lines have been purchased from the American Type Culture Collection. The original TRAMP-C2 and K562 cells, as well as the TRAMP-C2 and K562-based reporter transfects, have been routinely cultured in RPMI-1640 medium (Sigma) supplemented with 5% fetal bovine serum (Gibco) and 1% (v/v) penicillin/streptomycin cocktail (CellGro). The cells were subcultured 1:10 upon reaching near confluency or when detached. THP-1 and all related transfects were cultured in the RPMI-1640 supplemented with 10% heat-inactivated FBS (HyClone), additional 2 g/L glucose, 1 mM sodium pyruvate, 10 mM HEPES (both from Sigma), and 1% penicillin/streptomycin. The cell density was maintained between 1×10^5^ and 1×10^6^ cell/ mL. The standard culturing conditions for all cells were 37°C, 5% CO_2_, and 100% humidity.

### Plasmid constructs

Super *piggyBac* transposase expression vector and pB513B-1 transposon vector were purchased from System Biosciences (Mountain View, CA). 

pS**B**: Plasmid pB513B-1 was digested with *SwaI/EcoRV* restriction enzymes, and the smaller fragment was inserted into pSmart_HCKan (Lucigen, Middleton, WI) using the CloneSmart reaction.

pS**F**: The *pB* ITRs were obtained by PCR of pB513B-1 with the following primer sets. For 3’ITR: TCGTTAAATCGGATCGAACACGCAGCTAGATTAAC and TTGCGGCCGCGGATCCGATCTCGATATACAGACCGATAA. For 5’ITR : CAATACTGACCATTTAAATCGCTATTTAGAAAGAGAGAG and GGTCAGGTATGATTTACAGCTATGACCATGATTAC. Then pS**B** was digested with *BamHI/ SwaI* and both pieces isolated. These four fragments were assembled into pS**F** in a one-step In-Fusion reaction according to the manufacturer’s (Clontech) protocol. 

pS**A** and pS**E**: The 5’HS4 insulator was excised from pS**B** and pS**F** with *BstXI/ XbaI*, and the gap was In-Fusion-patched with a PCR product of pB513B-1 and the following primers: TTTTTTAGGGCCCATGTAATACGGTTATCCACAGA and TTGAGCGATATCTAGACTGGTATCTTTATAGTCCTGTC. 

pS**C**, pS**D**, pS**G**, and pS**H**: The 3’HS4 insulator was obtained by PCR of pB513B-1 with the following primers: ATCGGATCCGCGGCCGGTCTGTATATCGAGGTTTA and CAGATCCTTGCGGCCGCCATAATACTAGTAGGCCTTGG. It was brought into the In-Fusion reaction with NotI digests of pS**A**, pS**B**, pS**E**, and pS**F**, respectively.

pTR01F: The NF-κB pathway reporter sequence (Figure S4) has been amplified from pGL4.32 (Clontech) using the primers: TGGCAGTACATCTACGTAAACTAGCAAAATAGGCTGTC and CCGCGGATCCGATTTAGAAGGTAGCTAACCAAGTT. Next, P513B-1 was digested with *SnaBI/ SwaI*. The larger DNA fragment was then brought into the In-Fusion reaction with the reporter PCR product.

pTR03F: Two inserts containing 15-bp overhang sequences for the In-Fusion reaction and total 8 hypoxia response elements (Figures S4 and S9) were synthesized and annealed. The inserts and a product of pTR01F digestion with *NheI/ BglII* were assembled into pTR03F in one step following the In-Fusion protocol.

pTR05F: In a similar manner, two inserts containing the 15-bp overhang sequences and total 8 antioxidant/electrophile response elements (Figures S4 and S9) were synthesized and annealed. The inserts and the above pTR01F digest were assembled into pTR05F in one step using the In-Fusion reaction.

All constructs were confirmed by DNA sequence analysis.

### Transfections

TRAMP-C2 and K562 were seeded into wells of a 96-well plate, at 1×10^4^ cells per well in antibiotic-free RPMI-1640 supplemented with 5% FBS and left to adhere for 6 hours. The cells were then treated with 100 ng of reporter plasmid constructs complexed with TransIT Prostate reagent (Mirus) at 1:2 (μg/ μL) ratio, without the Boost reagent. See Table S1 for transfection efficiencies with this and other transfection reagents. In most cases, 33 ng Super *pB* transposase vector was co-transfected along with *pB* transposon plasmids. After 16 h, the regular media were added and the cells were left to proliferate for next 48 h. The transfected cells were then treated with a selecting antibiotic (100 μg/ mL G418 or 5 μg/ mL puromycin) for another week, and the surviving cells were expanded for cryopreservation and flow cytometry experiments.

Suspensions of 2×10^5^ THP-1 cells in 300 μL RPMI-1640, supplemented with 5% heat-inactivated FBS, were co-treated with 66 ng Super *pB* transposase plasmid and 200 ng of *pB* transposon reporter vectors complexed with GeneIn Transfection reagent set (Global Stem) at 1:8 (μg/ μL) ratio. After 16 h, the regular media were added and the cells were left to proliferate for next 48 h. Typical percentage of fluorescent cells at this point was, depending on the vector size, from 0.5 to 5%; see Table S1 for transfection efficiencies with this and other transfection reagents. The transfected cells were then treated with 5 μg/ mL puromycin for another week, and the surviving cells were expanded and frozen away.

Tranductions of TRAMP-C2 with Lenti GFP Control (Qiagen), at 1×10^5^ TU per well containing 1×10^4^ cells, were carried out according to the manufacturer’s protocol.

### THP-1 Differentiation

#### Into macrophages

Suspensions of the original THP-1 or the reporter lines in the differentiation serum-free medium CellGro Complete (Mediatech/ Corning) have been seeded at 2×10^4^ cells/well in 96-well plates. The cells were then treated with 10 ng PMA/well for 3 days, when all cells became adherent and assumed the macrophage morphology. The transformed cells were washed and kept in the RPMI-1640 (5% FBS, pen/strep) for a day, before any assays.

#### Into dendritic cells

The cells were seeded as above, using serum-free OptiMEM I (Gibco/ Life Technologies) as a differentiation medium and then treated with 10 ng GM-CSF and 20 ng IL-4 per well for 16 hours, followed by 2 ng TNFα and 20 ng ionomycin per well [31]. After 2 days, when most of adherent cells acquired DC appearance, the differentiated cells have been washed and kept in RPMI-1640 (5% FBS, pen/strep) containing reduced amounts of GM-CSF and IL-4 (2 and 4 ng per well, respectively), before any assays.

### Reporter activity assay

In a typical experiment, naïve or differentiated THP reporters seeded in 96-well plates were treated with sensitizers in 100 μL of serum-free CellGro Complete medium (Corning) for specific times in the standard cell culturing conditions (37°C, 5% CO_2_, 100% humidity). TRAMP-C2 and K562-derived reporters were seeded at 1×10^4^ or 2×10^4^ cells/well, respectively, in the culturing medium two days prior treatments. The assay medium was the complete RPMI-1640 less Phenol Red indicator. After the treatments, the reporter cells were washed and lysed in 60 μL of the Luciferase reporter lysing buffer (Promega). The lysates fluorescence was measured at the 482(9)/512(17) nm wavelength(width) setup, followed by addition of the luciferase substrate (Promega) and kinetic luminescence measurements in the wells, with 2-minute intervals, for 30 minutes total. All the measurements were done using a Synergy MX (BioTek) plate reader. The GFP fluorescence values were used for both evaluation of relative cell proliferation and normalization of the reporter luciferase activities in respective wells.

### Flow Cytometry

All experiments were done using a Beckman Coulter MoFlo XDP instrument. Normally, the instrument was calibrated with the Sphero Rainbow Beads (BD Biosciences). Analysis of the FACS data was performed with help of Summit 5.2 software package.

### Statistical Analysis

Statistical tests and plots were done using SigmaPlot software, version 11.0.

## Supporting Information

Figure S1 **Conventional transfections or viral transduction fail to provide stable expression of GFP in TRAMP-C2 cells.** TRAMP-C2 were **A**) transfected with a control pEGFP-C1 plasmid, **B**) transduced with Cignal LentiGFP, or **C**) co-transfected with PB513B-1 and *pB*ase. The cells then went through several rounds of fluorescence activated cell sorting in two-week intervals, by selecting 5% of the brightest (R4 cut on the histograms) cells for further proliferation. The respective fluorescence histograms are positioned in the columns and the sort rounds – in rows, from top to bottom. The cytometer settings were not uniformly calibrated on different days in this experiment. The fluorescence profiles of transfected/transduced TRAMP-C2 cells are shown in solid blue, while auto-fluorescence profiles of non-transfected cells are overlayed as contour lines.
(EPS)

Figure S2 **A representative view of the fluorescence “mosaic” distribution** in FAC-sorted (6 rounds) DU145 cells transfected with a control GFP plasmid.
(EPS)

Figure S3 ***pB*-based, insulated reporters are stably expressed in K562 line.** K562 cells were co-transfected with *pB* transposase and pS**A**, pS**D**, or pS**H** reporter plasmids. The transfects went through several rounds of FAC sorting, until the maximum fluorescence has been achieved (the upper histogram in each pair). The sorted cells were then let to proliferate without any selection and were subcultured twice a week. The lower histogram in each pair represents FACS analysis of the cells at the end of the 3-month term. The FACS instrument settings were uniformly calibrated against Sphero Rainbow Beads. Median fluorescence intensities were normalized for the non-transfected control K562 cell autofluorescence.
(EPS)

Figure S4 **Schematic representation of the pathway reporter constructs** , which are flanked by *pB* and cHS4 sequences as in **H** (Figure 1). The transcription response element, minimal CMV promoter, and firefly luciferase encoding sequences were inserted into p513B-1, resulting in pTR01F, pTR03F, and pTR05F plasmids. Sequences of TREs for HIF-1α and Nrf2 are given with essential nucleotides capitalized. TREs for NF-κB were cloned from pGL4.32.
(EPS)

Figure S5 **NF-κB reporter activity in TC2.R01F.** The cells were treated with proinflammatory agents, in triplicates, for 6 hours, then lysed. **A**. Fluorescence in the lysates was normalized against the control. **B**. Luciferase activities in the lysates were normalized against the respective fluorescence values and then against the control. The bars and error bars are normalized fluorescence or luminescence means ± SD, n = 3; statistical analysis: pairwise comparisons by an unpaired t-test. Statistical significance *: the lowest dose treatment compared to control; in all cases, *P*<0.001.
(EPS)

Figure S6 **HIF-1α reporter activity in TC2.R03F.** The cells were treated with hypoxia mimicking agents, in triplicates, for 15 hours, then lysed. **A**. Fluorescence in the lysates was normalized against the control. **B**. Luciferase activities in the lysates were normalized against the respective fluorescence values and then against the control. The bars and error bars are normalized fluorescence or luminescence means ± SD, n = 3; statistical analysis: pairwise comparisons by an unpaired t-test. Statistical significance *: the lowest dose treatment compared to control.
(EPS)

Figure S7 **Nrf2 reporter activity in TC2.R05F.** TC2.R05F cells were treated with electrophilic agents, in triplicates, for 14 hours, then lysed. **A**. Fluorescence in the lysates was normalized against the control. **B**. Luciferase activities in the lysates were normalized against the respective fluorescence values and then against the control. The bars and error bars are normalized fluorescence or luminescence means ± SD, n = 3; statistical analysis: pairwise comparisons by an unpaired t-test. Statistical significance *: the lowest dose treatment compared to control.
(EPS)

Figure S8 **NF-κB reporter activity in K562.R01F.** The cells were treated with proinflammatory agents, in triplicates, for 6 hours, then lysed. **A**. Fluorescence in the lysates was normalized against the control. **B**. Luciferase activities in the lysates were normalized against the respective fluorescence values and then against the control. The bars and error bars are normalized fluorescence or luminescence means ± SD, n = 3; statistical analysis: pairwise comparisons by an unpaired t-test. Statistical significance *: the lowest dose treatment compared to control; in all cases, *P*<0.05.
(EPS)

Figure S9 **Octamer transcription response element sequences for the In-Fusion mediated construction of pTR03F and pTR05F vectors, respectively.**
(EPS)

Table S1 **Efficiencies (%) of transient transfections of the cell lines after 48 hours.**
(DOCX)
