# Supplementary material for: piggyBac Transposon plus Insulators Overcome Epigenetic Silencing to Provide for Stable Signaling Pathway Reporter Cell Lines
Source: PLoS One. 2013 Dec 20;8(12):e85494. doi: 10.1371/journal.pone.0085494 (PMC3869926; doi:10.1371/journal.pone.0085494)
Supplement: Table S1 — Efficiencies (%) of transient transfections of the cell lines after 48 hours. (DOCX) [file pone.0085494.s010.docx]

**Table S1. Efficiencies (%) of transient transfections of the cell lines after 48 hours**. Indicated reporter vectors were premixed with the Super *piggyBac* transposase vector at the 3:1 ratio and then mixed with the transfection reagents in OptiMEM I medium to form the complexes according to the manufacturer’s protocols. Shown cDNA : reagent ratios were established as optimal in preliminary experiments. The cDNA/reagent complexes were added to cells in RPMI-1640 medium supplemented with 5 % heat-inactivated FBS, at the 100 ng cDNA per 100,000 (THP-1) or 10,000 (K562 or TRAMP-C2) cells load. After 48 hours, transfected cells, along with the non-transfected controls were lifted and subjected to FACS analysis for GFP fluorescence. The percentage of fluorescent cells was measured by arbitrarily setting the fluorescence intensity cut-off at 3.5 times over the controls median fluorescence for each cell line. The values in the Table represent averages of two independent transfections less the control average, ± range.

|  |  | Transfection reagent, cDNA : reagent (:boost) μg/μL ratio | | | | | |
| --- | --- | --- | --- | --- | --- | --- | --- |
| Cell line | Vector, size | FuGene HD 1:2 | GeneIn 1:8:8 | Lipofectamine 2000, 1:3 | Lipofectamine LTX, 1:1:1 | PolyMagNeo 1:2 | TransIT Express(*), 1:3 or Prostate(**), 1:2 |
| THP-1 | *pmaxGFP*, 4712 bp | 0.63 ±0.15 | 4.87 ±0.06 | 0.33 ±0.10 | 0.95 ±0.02 | 1.65 ±0.13 | 0.11 ±0.04 * |
|  | *pS****H***, 5616 bp | 0.51 ±0.03 | 3.80 ±0.15 | 0.14 ±0.05 | 0.47 ±0.04 | 0.35 ±0.13 | 0.34 ±0.06 * |
|  | *pTR01F*, 9319 bp | 0.25 ±0.03 | 0.86 ±0.12 | 0.10 ±0.04 | 0.11 ±0.03 | 0.23 ±0.12 | 0.09 ±0.05 * |
| K562 | *pTR01F* | 2.5 ±0.6 | 2.4 ±0.3 | 3.09 ±0.07 | 3.5 ±0.4 | 2.47 ±0.07 | 1.93 ±0.10** |
| TRAMP-C2 | *pTR01F* | 1.86 ±0.08 | 1.8 ±0.4 | 0.25 ±0.03 | 0.20 ±0.03 | 0.01 ±0.01 | 1.7 ±0.3 ** |
